# Adenoviromics: Mining the Human Adenovirus Species D Genome

**DOI:** 10.3389/fmicb.2018.02178

**Published:** 2018-09-11

**Authors:** Ashrafali M. Ismail, Ji Sun Lee, Jeong Yoon Lee, Gurdeep Singh, David W. Dyer, Donald Seto, James Chodosh, Jaya Rajaiya

**Affiliations:** ^1^Howe Laboratory, Massachusetts Eye and Ear, Harvard Medical School, Boston, MA, United States; ^2^Molecular Virology Laboratory, Korea Zoonosis Research Institute, Jeonbuk National University, Jeonju, South Korea; ^3^Department of Cell and Systems Biology, University of Toronto, Toronto, ON, Canada; ^4^Department of Microbiology and Immunology, University of Oklahoma Health Sciences Center, Oklahoma City, OK, United States; ^5^Bioinformatics and Computational Biology Program, School of Systems Biology, George Mason University, Manassas, VI, United States

**Keywords:** adenovirus, genome, evolution, transcription factor, interactome

## Abstract

Human adenovirus (HAdV) infections cause disease world-wide. Whole genome sequencing has now distinguished 90 distinct genotypes in 7 species (A-G). Over half of these 90 HAdVs fall within species D, with essentially all of the HAdV-D whole genome sequences generated in the last decade. Herein, we describe recent new findings made possible by mining of this expanded genome database, and propose future directions to elucidate new functional elements and new functions for previously known viral components.

## Introduction

Human adenovirus (HAdV) infections represent a significant source of morbidity and mortality, world-wide and at all ages, through highly transmittable infections at mucosal sites, including the eye, and urinary, respiratory, and gastrointestinal tracts (Horwitz, 1996). HAdV causes fatal acute respiratory distress syndrome in healthy adults and is especially lethal in infants and the immune compromised (Bhanthumkosol, 1998; Ryu et al., 2003; Wallot et al., 2006; Engelmann et al., 2016; Tan et al., 2016; Zhang et al., 2016). No FDA-approved therapy for acute HAdV infection is available. At resolution of acute infection, persistence may develop within nasopharyngeal lymphoid tissue (Neumann et al., 1987; Garnett et al., 2002, 2009; Zhang et al., 2010; Assadian et al., 2016), as yet uncharacterized cells in the gastrointestinal tract (Roy et al., 2009), and possibly the ocular surface (Kaye et al., 2005), permitting evolution of new HAdVs through homologous recombination between two or more HAdVs infecting the same cell(s) (Lee et al., 2005, 2018; Echavarria et al., 2006; McCarthy et al., 2009; Seto et al., 2010).

HAdVs are divided phylogenetically into seven species (A-G), with a total of 90 recognized genotypes with whole genome sequences in GenBank, including the original 51 “serotypes”—determined by serum neutralization—which now all have been fully sequenced (Table 1) (Robinson et al., 2013a) Human adenovirus species D (HAdV-D) is the largest and most rapidly growing among all HAdV species, and contains viruses associated with epidemic keratoconjunctivitis (EKC), a severe, hyperacute ocular surface infection (Butt and Chodosh, 2006). A collaboration funded by the American Recovery and Reinvestment Act of 2009 came to fruition with the complete whole genome sequencing and analysis of all previously unsequenced HAdV-D serotypes (Robinson et al., 2013a), leading to a new understanding of adenovirus ontogeny (Jones et al., 2007; Robinson et al., 2008, 2009a,b; Robinson et al., 2011b,c; Robinson et al., 2013a,b; Walsh et al., 2009, 2010a,b; Arnold et al., 2010; Torres et al., 2010; Dehghan et al., 2011, 2013a,b; Walsh et al., 2011; Liu et al., 2011; Seto et al., 2011, 2013; Singh et al., 2012, 2013; Zhou et al., 2012)—including those HAdV-Ds associated with EKC (Robinson et al., 2008, 2009b, 2011b; Walsh et al., 2009; Zhou et al., 2012)—and ultimately to a new typing system for HAdV based on genomics (Seto et al., 2011).

**Table 1:** Species and type designations for the 51 human adenovirus (HAdV) serotypes.

**Type**	**GenBank accession no**.	**Genome length**	**Year published**
HAdV-C1	AC_000017	36001	2004
HAdV-C2	AC_000007	35937	2003
HAdV-B3	AY599834	35345	2006
HAdV-E4	AY599837	35964	2006
HAdV-C5	AY601635	35931	2006
HAdV-C6	FJ349096	35758	2011
HAdV-B7	KP670856.2	35239	2016
HAdV-D8	AB448767	34980	2009
HAdV-D9	AJ854486	35083	2008
HAdV-D10	JN226746	35105	2013
HAdV-B11	AF532578	34794	2003
HAdV-A12	X73487	34125	1979
HAdV-D13	JN226747	35209	2013
HAdV-B14	JQ824845	34767	2012
HAdV-D15	KF268204	35100	2013
HAdV-B16	JN860680	35384	2011
HAdV-D17	HQ910407	35139	2011
HAdV-A18	GU191019	34177	2010
HAdV-D19	JQ326209	35153	2011
HAdV-D20	JN226749	35181	2013
HAdV-B21	AY601633	35382	2006
HAdV-D22	FJ619037	35152	2009
HAdV-D23	JN226750	35050	2013
HAdV-D24	JN226751	35166	2013
HAdV-D25	JN226752	35248	2013
HAdV-D26	EF153474	35152	2007
HAdV-D27	JN226753	35154	2013
HAdV-D28	FJ824826	35130	2010
HAdV-D29	JN226754	35214	2013
HAdV-D30	JN226755	35178	2012
HAdV-A31	AM749299	33763	2005
HAdV-D32	JN226756	35248	2013
HAdV-D33	JN226758	35131	2013
HAdV-B34	AY737797	34775	2004
HAdV-B35	AC_000019	34794	2004
HAdV-D36	GQ384080	35152	2010
HAdV-D37	AB448775	35215	2009
HAdV-D38	JN226759	35221	2013
HAdV-D39	JN226760	35152	2013
HAdV-F40	NC_001454	34214	1993
HAdV-F41	DQ315364.2	34188	2007
HAdV-D42	JN226761	35231	2013
HAdV-D43	JN226762	35012	2013
HAdV-D44	JN226763	35214	2013
HAdV-D45	JN226764	35154	2013
HAdV-D46	AY875648	35178	2006
HAdV-D47	JN226757	35106	2013
HAdV-D48	EF153473	35206	2007
HAdV-D49	DQ393829	35215	2006
HAdV-B50	AY737798	35385	2007
HAdV-D51	JN226765	35114	2013

Recent published work demonstrates how genome “mining,” in-depth analyses of the growing HAdV genome database, can bring about new realizations and add critical new information to prior ones. The trimeric fiber protein on adenoviruses mediates viral entry through interaction of the distal most “knob” structure on the fiber with host cell receptors. In a phylogenetic analysis of HAdV-D fiber genes, HAdV-D types associated with EKC were recently shown to form a unique clade (Ismail et al., 2016). By proteotyping, a new *in silico* methodology described in detail below, EKC virus-associated fiber knobs were uniquely shared, and signature amino acid positions distinguished EKC from non-EKC types. Remarkably, human corneal epithelial cell tropism could be predicted by the presence of a lysine or alanine at residue 240, and this amino acid residue in EKC viruses showed evidence for positive selection. These data added to the prior observation by Huang and coworkers that artificial mutation to a lysine at residue 240 in a non-EKC virus could confer infection of Chang cells, a conjunctiva derived continuous cell line (Huang et al., 1999). However, because Chang cells came later known to be contaminated by HeLa cells, the importance of residue 240 to ocular tropism was until this new observation, in some doubt.

**Figure 1:**
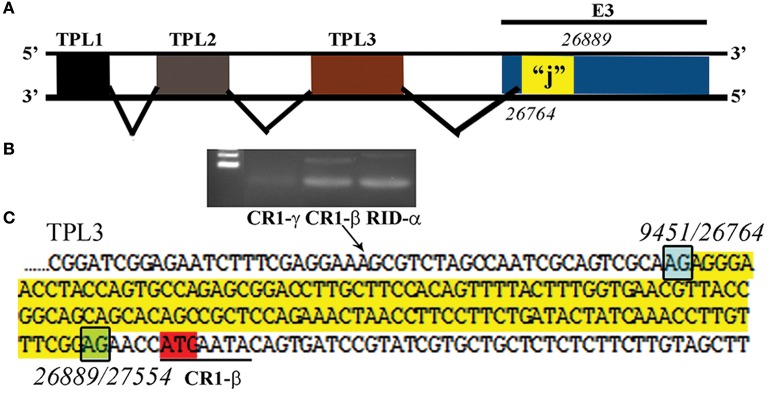
Putative “j”-leader located within the CR1-α E3 gene. **(A)** Schematic for the location of a newly detected leader (“j”-leader) embedded within the E3 CRI-α gene, experimentally determined to be spliced to some, but not all mRNAs of the E3 genes. **(B)** Gel photomicrograph of mRNA transcripts amplified with forward primer from TPL1 and reverse primers from CR1-γ, CR1-β, and RID-α. Primers were chosen to elicit similarly sized bands to facilitate subsequent sequencing. **(C)** Nucleotide sequence of the PCR product for CR1-β. The putative j-leader sequence and splice sites are shown in yellow and green, respectively. Note an additional 4 nucleotide 5′UTR (AACC) prior to the CR1-β start site (red). The 5′UTR in **(C)** prior to the splice site for the j-leader is from TPL3. Adapted from Ramke et al. (2017) with permission.

Another recently published effort provided further evidence of the importance and potential for HAdV genome mining. Late adenoviral gene expression is initiated by the adenovirus major late promoter (Ramke et al., 2017), followed by splicing of mRNAs to the viral tripartite leader for translation (Chow et al., 1977; Akusjärvi and Pettersson, 1978; Chow and Broker, 1978; Logan and Shenk, 1984). The HAdV tripartite leader is a 200-nucleotide 5' noncoding region that circumvents the requirement for eukaryotic initiation factor 4F or cap binding protein complex (Ziff and Evans, 1978; Akusjärvi and Pettersson, 1979; Dolph et al., 1988; Zhang et al., 1989), and permits translation of HAdV mRNAs at late times in infection when cap-dependent translation is blocked due to shut down of host cellular cap-dependent mRNA translation. HAdV 5′ untranslated regions (5′UTRs) are critical for cap-independent initiation, and impact mRNA localization and stability. The HAdV tripartite leader (TPL), composed of three introns (TPL 1-3), drives translation of HAdV late mRNA. The annotation of 72 HAdV genotypes for the HAdV TPL and another previously described leader, the i-leader, let to identification of newly identified polycistronic mRNAs for RID-α and RID-β within the E3 transcription unit, and a potential new open reading frame (ORF) within the i-leader sequence, with termination of this potential protein in TPL3 (Ramke et al., 2017). In addition, the authors also identified a potential new leader sequence embedded within the E3 region, tentatively named the j-leader (Figure 1).

## Structure and infection

The HAdV is non-enveloped, icosahedral in shape, and contains a double stranded DNA genome of ~36,000 base pairs (bp) with ~1 open reading frame (ORF) for every 1000 nucleotides. Viral DNA is associated with four (interior) core proteins including Mu, VII, V, and terminal protein. The histone-like protein (p) VII protects viral DNA from cellular DNA damage responses (Lischwe and Sung, 1977; Karen and Hearing, 2011; Avgousti et al., 2017). The outer protein coat (capsid) of the virus consists of 240 hexon capsomers and 12 penton capsomers, along with several minor capsid proteins. The latter include pVI, pIIIa, pVIII, and pIX and are important to capsid stability. Each penton capsomer contains a ring of five penton base proteins which bind and support the trimeric fiber protein with its distal fiber knob. During viral infection, the fiber knob binds to one of several host cell receptors (Nemerow, 2000; Goosney and Nemerow, 2003; Nemerow et al., 2009). The penton base protein contains two hypervariable loops. The interaction between fiber knob and a host cell receptor brings about secondary contact between the hypervariable loop 2 (HVL2) arginine-glycine-aspartic acid (RGD) motif in each penton base protein (five per penton base capsomer) with host cell integrins α_v_β_3_, α_v_β_5_, and α_v_β_1_, that in turn induce endocytosis of the virus (Li et al., 1998a,b; Li et al., 2000). HAdV structural proteins can serve multiple functions. For example, the minor capsid structural protein VI (pVI) plays a critical role in at least three distinct aspects of the viral “life” cycle: endosomal escape during cell entry, nuclear assembly during viral replication, and stability of the intact, infectious virus outside the host (Wodrich et al., 2003; Wiethoff et al., 2005; Moyer et al., 2011, 2016). These findings suggest that, as with pVI, other HAdV structural proteins may have multiple functions yet to be elucidated.

## Genomics and evolution

**Figure 2:**
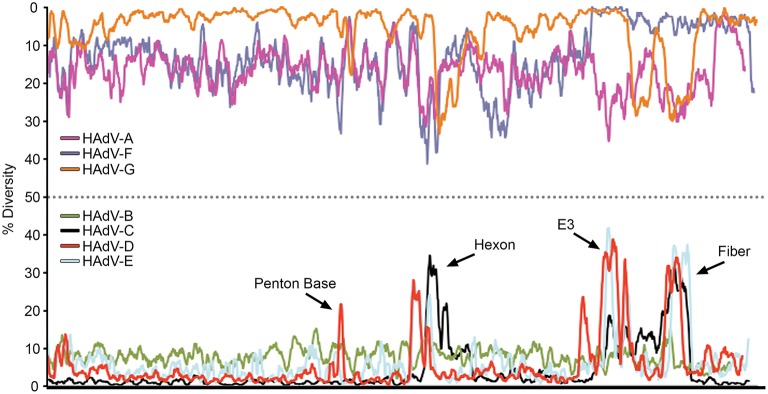
Nucleotide diversity plots, by HAdV species, generated with DnaSP, represent the average number of nucleotide differences per site between each type in every HAdV species. The % diversity is calculated on the y-axis; the x-axis illustrates the nucleotide position on the genome. HAdV-Ds (red line on bottom half of plot) show particular diversity in the penton base, hexon, E3, and fiber coding regions, with otherwise very high conservation. From Robinson et al. (2013a) with permission.

**Figure 3:**
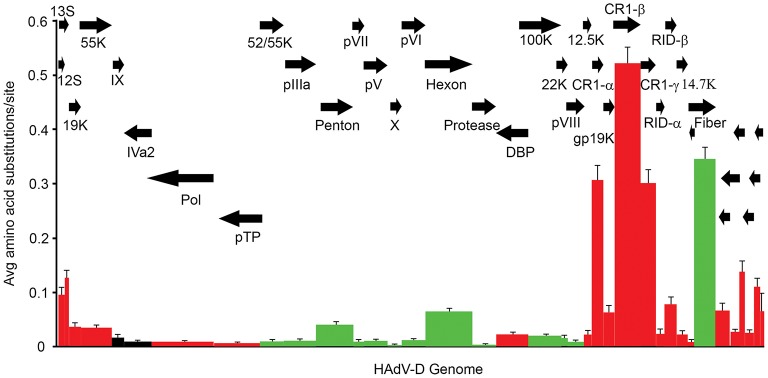
Amino acid diversity calculated in MEGA 4.02, measuring the average amino acid substitution for each HAdV-D protein. Each bar in the graph corresponds to a protein as represented by arrows. Red = early genes. Dark green = late genes. Black = intermediate genes. The hypervariable loops of penton base and hexon proteins were also analyzed separately (light green) and showed particularly high levels of amino acid substitutions. From Robinson et al. (2013a) with permission.

The relatively large genome database for HAdV-D (over 50 unique viruses with available whole genome sequences) (Tables 1, 2) has permitted detailed analyses of genome relationships within this clinically important adenovirus species. HAdV-D genomes are highly conserved (>90%). However, whole genome analyses of HAdV-D have revealed specific loci of genetic hypervariability in the hexon, penton base, fiber, and E3 CR1α, β, and γ genes (Figure 2), dictating nonsynonymous amino acid changes in corresponding proteins (Figure 3). GC content confers genome stability and resistance to recombination (Gruss et al., 1991), and the genomes of HAdV-D have among the highest GC content among HAdV species (~56%). The hypervariable regions in HAdV-D were found to be sharply reduced in GC nucleotide content relative to the rest of the genome (Robinson et al., 2013a). Mutations in HAdV are relatively infrequent, with genome stability now documented in some types across decades (Hofmayer et al., 2009; Mahadevan et al., 2010; Seto et al., 2010; Dehghan et al., 2013b; Robinson et al., 2013a; Alkhalaf et al., 2015). However, those regions of the genome shown to be hypervariable and relatively low in GC content are the very same also shown to undergo homologous recombination (Robinson et al., 2009a, 2011b; Walsh et al., 2009; Zhou et al., 2012; Singh et al., 2013), driving the evolution of new genotypes.

**Table 2:** Species and molecular types of human adenovirus (HAdV) genotypes 52–90.

**HAdV type**	**^#^Name**	**GenBank accession no**.	**Genome length**	**Year published**
HAdV-G52	P52H52F52/2003/USA	DQ923122.2	34250	2007
HAdV-D53	P37H22F8/2005/DEU	FJ169625	34909	2009
HAdV-D54	P54H54F8/2000/JPN	AB333801	34920	2008
HAdV-B55	P14H11F14/2006/CHN	FJ643676	34755	2010
HAdV-D56	P56H15F9/2008/FRA	HM770721	35066	2011
HAdV-C57	P1H57F6/2001/RUS	HQ003817	35818	2011
HAdV-D58	P58H58F29/1996/ARG	HQ883276	35217	2011
HAdV-D59	P64H25F56/2007/USA	JF799911	35072	2012
HAdV-D60	P60H20F60/2009/CAN	HQ007053	35050	2013
HAdV-A61	P31H31F31/2004/JPN	JF964962	33776	2011
HAdV-D62	P62H62F62/1993/GBR	JN162671	35127	2014
HAdV-D63	P30H30F29/1959/USA	JN935766	35168	2012
HAdV-D64	P22H19F37/1993/USA	EF121005	35231	2012
HAdV-D65	P58H10F9/2004/BGD	AP012285	35172	2012
HAdV-B66	P66H7F3/1987/ARG	JN860676	35080	2012
HAdV-D67	P67H9F67/2005/BGD	AP012302	35075	2013
HAdV-B68	P16H3F16/2004/ARG	JN860678	35538	Unpublished
HAdV-D69	P53H15F69/1955/SAU	JN226748	35124	2013
HAdV-D70	P70H70F29/2014/DEU	KP641339	35186	2015
HAdV-D71	P9H20F71/1987/DEU	KF268207	35192	2013
HAdV-D72	P72H30F72/1985/DEU	KF268335	34553	2013
HAdV-D73	P67H45F27/2015/DEU	KY618676	35190	2017
HAdV-D74	P70H74F51/2015/DEU	KY618677	35155	2017
HAdV-D75	P75H26F29/2015/DEU	KY618678	35104	2017
HAdV-B76	P21H21F16/DEU	KF633445	35586	2013
HAdV-B77	P35H34F7/1985/DEU	KF268328	34653	2013
HAdV-B78	P11H11F7/2000/ARG	KT970440	34881	Unpublished
HAdV-B79	P11H34F11/2015/JPN	LC177352	34779	2017
HAdV-D80	P19,23H28F22/2014/DEU	TBA	34909	Unpublished
HAdV-D81	P65H48F60/2012/JPN	AB765926.1	35198	2014
HAdV-D82	P56H15F37/2011/JPN	LC066535.1	35122	Unpublished
HAdV-D83	P83H9F15/2010/PAR	KX827426.1	35207	2017
HAdV-D84	P43H17F84/2011/PAN	MF416150	35257	2017
HAdV-D85	P37H19F8/2015/JPN	LC314153	35203	2018
HAdV-D86	P9H25F25/1978/SWE	TBA	35147	Unpublished
HAdV-D87	P9H15F25/1967/USA	MF476841	35159	Unpublished
HAdV-D88	P88H15F9/1963/USA	MF476842	35115	Unpublished
HAdV-C89	P89H2F2/2015/DEU	TBA	35998	Unpublished
HAdV-D90	P33H27F67/2017/BGD	TBA	34207^*^	Unpublished

# Name indicates molecular type (P, penton base; H, hexon; F, fiber)/year of isolation/country of isolation.

TBA: GenBank accession number, to be assigned.

* *Metagenomics project, missing the inverted terminal repeat sequences*.

Adenoviruses recombine specifically during viral replication (Williams et al., 1975; Meinschad and Winnacker, 1980; Munz et al., 1983), and do so by both homologous and heterologous mechanisms (Young et al., 1984; Epstein and Young, 1991; Crawford-Miksza and Schnurr, 1996). However, the evidence for homologous recombination as the major mechanism driving HAdV-D evolution is unassailable (Robinson et al., 2013a; Singh et al., 2013). Specifically, recombination occurs in the two penton base hypervariable regions (these code for two hypervariable loops (HVLs) on the penton base protein, separated from one another by ~125 conserved amino acids), seven hexon hypervariable regions (these are closely adjacent in the hexon gene and determine two adjacent HVLs on the hexon protein), fiber (fiber gene and protein are entirely hypervariable), and E3 CR1α, β, and γ (each also entirely hypervariable). For homologous recombination between two HAdVs to occur, at least two virus types with high nucleotide sequence homology at corresponding locations in both genomes must co-infect the same cell, and viral DNA replication should be ongoing. Co-infection by two or more HAdVs has been well documented (Lee et al., 2005; Echavarria et al., 2006; Vora et al., 2006; McCarthy et al., 2009; Halstead et al., 2010; Seto et al., 2010), as has the presence of two HAdV types in archived clinical samples (Singh et al., 2012).

**Figure 4:**
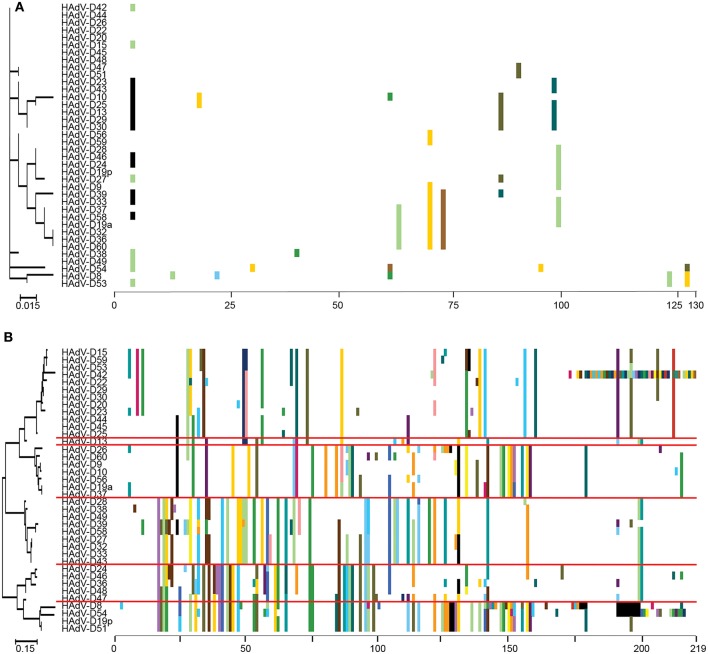
Proteotyping analysis comparing the HAdV-D E3 14.7K **(A)** and CR1α **(B)** proteins. The 14.7K protein was conserved, while CR1α demonstrated 6 unique proteotypes. Maximum likelihood phylogenetic trees are shown to the left for each putative protein, and amino acid signatures to the right. The scale bar at the bottom left of each sub-figure denotes the phylogenetic distance reflected in horizontal dimension of the respective tree. To construct the amino acid signatures shown, each amino acid was assigned a unique color (upper right corner), consensus amino acids at each position across all 38 viruses were assigned white, and gaps in the alignment were colored black. Horizontal red lines delineate distinct proteotypes. Adapted from. Singh et al. (2013) with permission.

“Proteotyping” is a novel approach to the study of genome evolution (Obenauer et al., 2006), and has been applied to characterize recombination among HAdV-D (Robinson et al., 2013a; Singh et al., 2013). In this method, maximum likelihood trees are used to align amino acid sequences of hypervariable, frequently recombined proteins. Each amino acid is assigned a unique, arbitrary color. Consensus residues are colored white, and gaps in the alignment are colored black. A threshold of <10% sequence divergence is used to distinguish unique proteotypes. An example of proteotyping is shown (Figure 4), comparing an amino acid alignment from E3 14.7K, a highly conserved gene with one distinct proteotype, with the hypervariable E3 CR1α (Singh et al., 2013), with six distinct proteotypes observed among 38 HAdV-Ds. E3 14.7K is therefore not hypervariable and not recombinant. E3 CR1α is hypervariable and recombinant.

**Figure 5:**
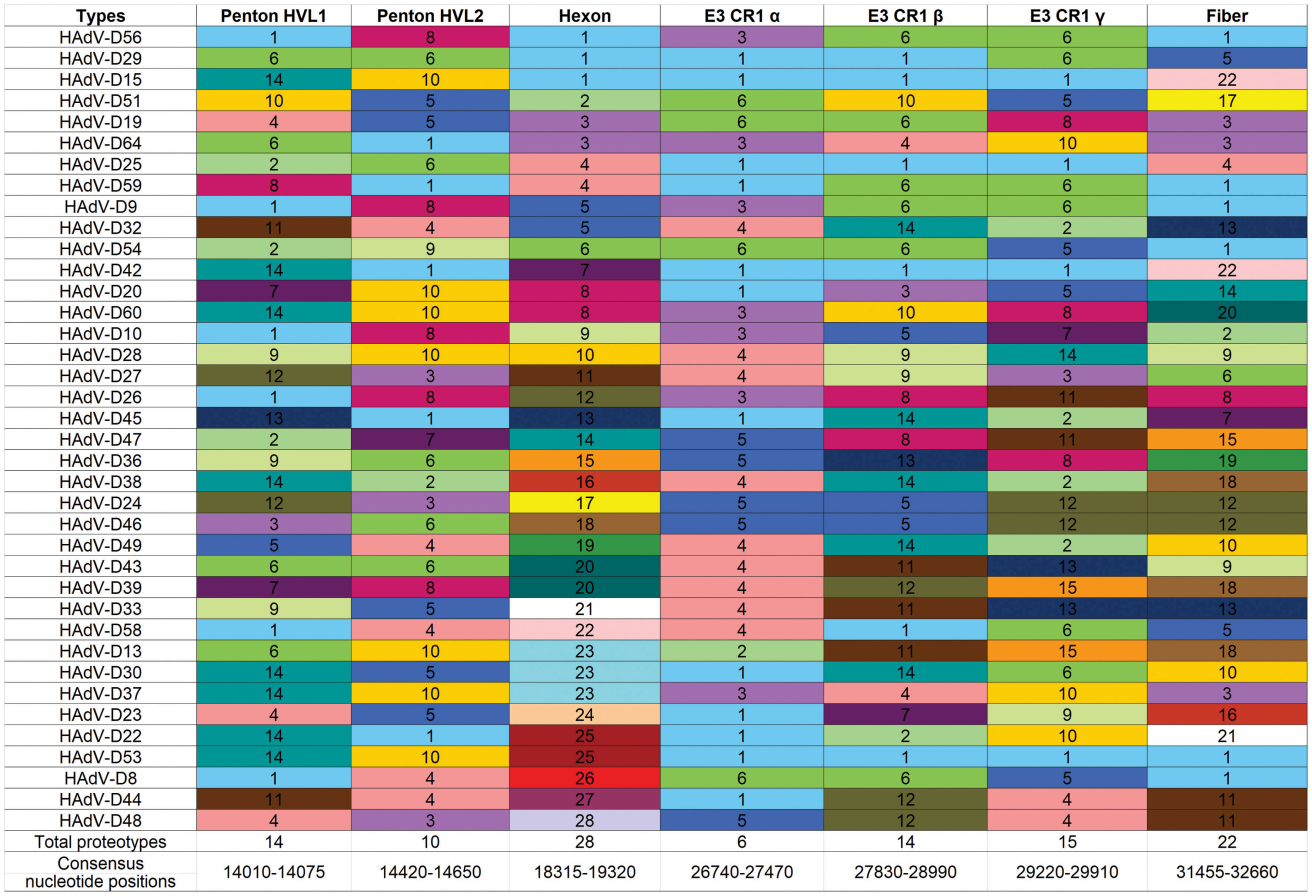
Proteotyping for 38 HAdV-Ds, sorted for the hexon proteotype column. Numbers and colors are arbitrary, and distinguish distinct proteotypes. Recombinants can be identified by rows. For example, HadV-D56, -D29, and -D15 fall within the same proteotype and are predicted to share highly similar nucleotide sequences for their respective hexon hypervariable regions (as confirmed by Singh et al., 2015). For HAdV-D29 and –D15, the recombination event extended through the E3 CR1β ORF gene and then ended.

Another way to interpret the analyses for those proteins like E3 CR1α, with more than one proteotype is that those proteotypes containing more than one HAdV type have previously recombined in nature, while those proteotypes with only one HAdV type are those that have not (yet) been shown to recombine in nature. HAdV-D37 and 29 fall within different hexon proteotypes (Figure 5). HAdV-D37 shares a hexon proteotype with HAdV-D13 and 30 (Robinson et al., 2013a), while HAdV-D29 shares a hexon proteotype with HAdV-D15, 56, and 69 (Singh et al., 2015). These two hexon proteotypes therefore have undergone prior homologous recombination. In contrast, the hexon proteins of HAdV-D10 and 28 are each in a proteotype with only one member; hexon recombination for these two viruses has therefore not yet been documented in nature. In sum, these data show by independent means that homologous recombination within HAdV-D is common, and confirm previously recognized patterns of homologous recombination among HAdV-D (Robinson et al., 2009a, 2011b, 2013a,b; Walsh et al., 2009, 2010a; Singh et al., 2012, 2013; Zhou et al., 2012; Gonzalez et al., 2014).

The local sequence and/or structure of DNA in regions flanking recombinogenic sites is significant for directing cellular recombination machinery to those regions. In bacteria, a signal for recombination between homologous DNA is the *c*rossover *h*otspot *i*nstigator, or Chi nucleotide sequence. This was first discovered in bacteriophage lambda, then in bacterial DNA, and later shown to mediate recombination between them (Stahl, 1998). The Chi sequence in *E. coli* (Chi_EC_) is 5′-GCTGGTGG-3′ (Smith et al., 1981; Bianco and Kowalczykowski, 1997), and its presence induces the conversion of the RecBCD enzyme from a helicase to an exonuclease, producing ssDNA that can invade homologous dsDNA during recombination (Taylor et al., 1985). The RecA enzyme of *E. coli* is loaded onto unwound ssDNA by RecBCD and promotes ssDNA exchange/recombination with homologous dsDNA (Cox, 1999; Smith, 2012). RecA has significant homology to eukaryotic Rad51 and its paralogs (Suwaki et al., 2011), enzymes that repair dsDNA breaks in human cells, facilitate homologous recombination, and during adenovirus infection, bind to the E2 DNA binding protein (Tookman et al., 2016). In our study of the region just 5′ to HVL2 on the penton base gene, a recombination hot-spot for HAdV-D (Robinson et al., 2009a), we found Chi-like sequences (Chi_AD_), e.g., 5′-ACTTCTGA-3′ in the proteotype containing HAdV-D64, and 5′-TCTCCTGA-3′ in the proteotype including HAdV-D37 (Lee et al., 2018). The putative Chi_AD_ sequences we identified in HAdV-D were found within the GC-rich component of GC/AT transition zones that precede and include HVL2, and were conserved within each proteotype. *In vitro, E. coli* lysates containing RecA protein increased recombination of two HAdV-D genotypes with the same penton base HVL2 proteotype. RecA was shown by ChIP to bind specifically to Chi_AD_ nucleotide sequence in the same regions, and also colocalize with adenovirus DNA within infected cell nuclei. These data suggest that Chi-like nucleotide sequences adjacent to the junction of conserved and hypervariable gene segments in HAdV-D may be an important signal for homologous recombination, and provide evidence in support of the idea that local bacterial flora might enhance natural recombination through Chi-like nucleotide sequences at HAdV-D recombination hotspots.

Another explanation for homologous recombination between HAdV, not exclusive of a role for Chi_AD_, is the potential for GC-low (AT-rich) single stranded DNA (ssDNA) to form hairpin loops (Nagy and Bujarski, 1997; Ohshima et al., 2007), a physical nonlinearity that would facilitate binding of ssDNA of one HAdV-D type to a homologous segment of ss or dsDNA from a physically adjacent but different HAdV-D type during co-infection of the same cell. Hairpin loops and other alterations in the physical configuration of ssDNA during DNA replication might also contribute to polymerase jumping (Jennings et al., 1983; Spaan et al., 1983; Pääbo et al., 1990; Viswanathan et al., 1999), in which physical constraints to polymerization lead to translocation of the DNA polymerase to an adjacent DNA from a different virus, resulting in a recombined DNA. Polymerase jumping has been shown to occur during HAdV DNA replication (King et al., 1997; de Jong et al., 2003), although it has not been suggested previously as a mechanism for HAdV-D evolution. Analysis of 38 HAdV-D whole genome sequences identified instances of 15 nucleotide-long GC-rich sequence adjacent to 15 nucleotide-long AT-rich sequence (sometimes with a 15 or 30 nucleotide-long GC-moderate sequence intervening), located just 5′ and 3′ to frequently recombined gene segments, and which were shown by *in silico* analysis of their corresponding ssDNA to form hairpin loops (Robinson et al., 2013a). Taken together, these data suggest covariant effects of nucleotide sequence and ssDNA secondary structures on homologous recombination between two HAdV-Ds.

## Transcriptome

**Figure 6:**
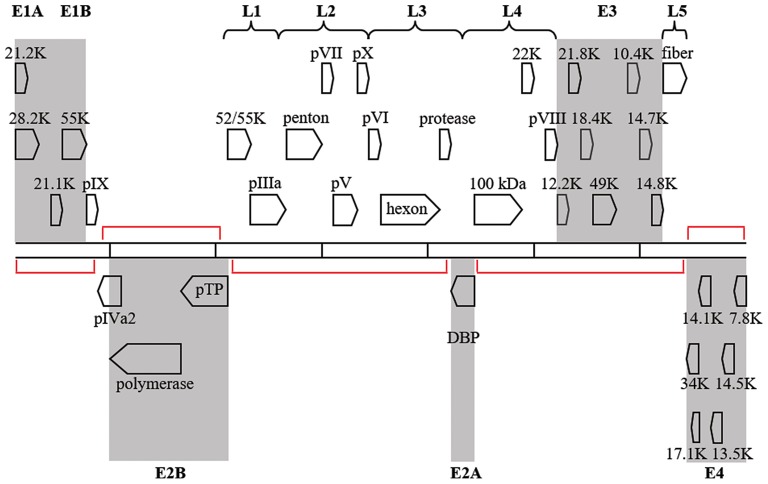
Transcription map for HAdV-D37. Genes are divided by early (shaded) or late expression. Red brackets denote large areas of “noncoding” DNA, but many additional, smaller, potential coding regions exist between and within known genes. Adapted from Robinson et al. (2008) with permission.

Regions of the HAdV-D genome currently thought to be “noncoding,” may contain functional elements. Because viruses exist on the nano-scale, viral genomes are by necessity constrained by size, and “junk” nucleotide sequences represent an extravagance. The National Human Genome Research Institute project to identify functional elements in the human genome (Encyclopedia of DNA Elements, or ENCODE) identified functionality in much of the human genome previously without known utility (Consortium et al., 2007; Qu and Fang, 2013; Kellis et al., 2014) The double-stranded DNA genomes of HAdV also contain regions with no known function. Transcriptional profiling of host gene expression has been studied after HAdV infection (Dorer et al., 2011) However, although viral transcriptomes have been reported for several viruses, most notably dengue, varicella zoster, and Epstein-Barr viruses (Ortmann et al., 2008; Ertl et al., 2011; Nagel et al., 2011, 2013; Arvey et al., 2013; Sujayanont et al., 2014), a *de novo* HAdV transcriptome has not been reported. Wu and coworkers used deep RNA sequencing to confirm known bat AdV transcripts (Wu et al., 2013), but did not investigate “noncoding” regions. *In silico* ORF prediction in HAdV can be difficult due to splice variants and inconsistencies in banked gene annotations (Davison et al., 2003), but in a prior annotation of HAdV-D37, ~60 new additional ORFs were predicted using in combination, the NCBI ORF finder, TIGR annotation engine, and GeneMark Heuristic model (Robinson et al., 2008) Putative genes were found within the large regions of noncoding DNA on the complementary strand opposite to established HAdV genes (Figure 6), in smaller regions on the coding strand within established transcription units but between confirmed genes, and overlapping or completely within established genes. Work is in progress in our laboratories to identify putative new genomic elements in HAdV by high-throughput sequencing of the viral transcriptome of HAdV-D37.

## Transcription factor binding sites

HAdV uses host TFs nuclear factor I and III (NF-I and NF-III) as part of the viral DNA replication complex (Pruijn et al., 1988; Mul et al., 1990; Hatfield and Hearing, 1991, 1993) Simian AdVs typically lack the NF-I binding site, while human viruses express it. It was previously reported that HAdV-E4, originally isolated in 1952, is a product of recombination between HAdV-B16 and the simian AdV, SAdV-E26. Clinical strains of HAdV-E4 isolated recently contain a NF-I binding site in the inverted terminal repeat (Houng et al., 2006; Dehghan et al., 2013a,b) that is absent in the original isolate (Purkayastha et al., 2005), suggesting that NF-I binding may be important to viral fitness in humans. To further elucidate mechanisms of viral gene expression, we are exploring novel TF binding sites on HAdV-D DNA, using ENCODE validated methodologies (Gerstein et al., 2012; Landt et al., 2012).

## Protein interactomes

The major HAdV capsid structural proteins—hexon, penton base, and fiber—interact directly with extracellular mediators of host immunity (Gahéry-Ségard et al., 1998; Molinier-Frenkel et al., 2002; Schoggins and Falck-Pedersen, 2006; Tamanini et al., 2006; Kalyuzhniy et al., 2008; Chintakuntlawar et al., 2010; Bradshaw et al., 2012; Flatt et al., 2013). The hexon, penton base, and fiber proteins also exhibit distinct amino acid signatures, characterizing discrete proteotypes (Robinson et al., 2013a). Gene products from the E3 transcription unit of HAdV function in viral immune evasion (Horwitz, 2004; Lichtenstein et al., 2004b; Windheim et al., 2004). In HAdV-D, the open reading frames for three of eight E3 genes—CR1α, CR1β, and CR1γ–are uniquely hypervariable compared to the other ORFs within the E3 transcription unit, and also segregate into discrete proteotypes (Singh et al., 2013). Highly conserved genes, such as DNA binding protein, DNA polymerase, and E3 14.7K, show no such variability (Robinson et al., 2013a; Singh et al., 2013).

While it may be assumed that hypervariablity in major capsid and E3 proteins is driven through evolutionary selection by the *extracellular* interactome, amino acid differences in a hypervariable protein can also lead to differences in that protein's *intracellular* interactome, the set of intrinsic host cell proteins which network with the viral protein, as was recently confirmed for E3 CR1 genes across HAdV species (Martinez-Martin et al., 2016). Viral capsid structural proteins are critical to virion stability. For the nonenveloped HAdV, fiber and penton base proteins on the external surface of the capsid serve as ligands for attachment to the host cell (Huang et al., 1999) and initiate viral entry (Wickham et al., 1993), respectively. HAdVs are typically internalized via endosomes. Endosomal acidification leads to structural instability of the capsid and endosomal release into the cytosol. HAdV capsid is then transported by microtubules to the nuclear membrane. Viral DNA then enters the nucleus through nuclear pores, leaving almost all the viral structural proteins in the cytosol (Henaff et al., 2011). Viral capsid proteins within the cell are eventually targeted for ubiquitination (Ko et al., 2010; Marvin and Wiethoff, 2012; Horan et al., 2013; Li et al., 2013) and degraded (Greber et al., 1993), but there are many opportunities for interaction with intracellular host cellular proteins during entry, trafficking, translation, assembly, and egress. Penton base HVL2, with its RGD motif, is critical to viral internalization through the interaction with host cell integrins,; (Wickham et al., 1993) but function of penton base HVL1 is unknown, and might be revealed though knowledge of its protein interactome. The closely adjacent hexon HVL1 and 2 form the epsilon epitope that determines serum neutralization, and interactions between the hexon protein and serum coagulation factor X confers liver tropism to HAdV-C5 (Sumarheni et al., 2014). However, nothing is known about potential hexon interactions with intracellular proteins during infection.

**Figure 7:**
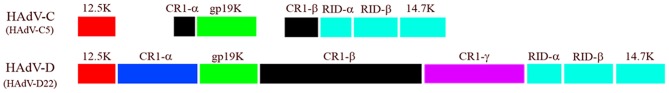
Comparison of E3 transcription unit from HAdV-C and -D. Note in particular the difference in ORF size between CR1β of the two HAdV species. Adapted from Robinson et al. (2011c) with permission.

The E3 transcription unit of HAdV codes for proteins that mediate immune evasion by the virus (Horwitz, 2004). Although E3 is labeled as an early transcription region, its transcripts are expressed both early and late during viral infection (Chow et al., 1977; Chow and Broker, 1978; Bhat and Wold, 1986), and there is evidence for at least one E3 protein that late transcripts are translated (Robinson et al., 2011a). E3 gene products are not required for viral replication in cultured cells (Morin et al., 1987), but inhibit cellular and cytokine mediated host immune responses to infection (Horwitz, 2004; Lichtenstein et al., 2004b; Windheim et al., 2004). Almost all of what is known about the function of specific E3 proteins derives from studies on HAdV-C. For example, HAdV-C2 E3 CR1α directs another E3 protein (19K) to the endoplasmic reticulum of cytotoxic T cells (Wilson-Rawls et al., 1994), where 19K binds to and retains MHC class I proteins (Jefferies and Burgert, 1990), preventing presentation of viral peptides within MHC class I at the cell surface (Burgert and Kvist, 1985, 1987; Andersson et al., 1987; Burgert et al., 1987; Cox et al., 1991). CR1α, RIDα, and RIDβ proteins cooperate to evade TNFα-related apoptosis through TRAIL (Elsing and Burgert, 1998; Tollefson et al., 1998; Benedict et al., 2001; Lichtenstein et al., 2004a). CR1β (Wold et al., 1984), also called the adenovirus death protein (Tollefson et al., 1992), is required for cell lysis (Tollefson et al., 1996) and viral spread (Doronin et al., 2003). The ORF size of each E3 gene varies across HAdV species (Figure 7) (Robinson et al., 2011c). Similarly, immune evasion functions of E3 gene products may not be the same across HAdV species, or function similarly in all cell types (Routes and Cook, 1990). Windheim and coworkers recently showed that the CR1β protein of the eye pathogen HAdV-D64 suppresses natural killer cell function (Windheim et al., 2013). The E3 CR1 genes are uniquely hypervariable within HAdV-D, and as predicted, overlapping but distinguishable intracellular interactomes across proteotypes were recently reported by Martinez-Martin and colleagues, who used protein microarrays to identify novel CR1β binding partners (Martinez-Martin et al., 2016).

## Conclusions

HAdV was critical to the dual discoveries of viral oncogenesis and RNA splicing (Berget et al., 1977; Chow et al., 1977; Whyte et al., 1988). HAdV is also a significant agent of disease for which there is no approved treatment. Recent mining of HAdV genomes has been highly productive, and there is ample evidence to suggest that further whole genome analyses will elucidate new and fundamental mechanisms in HAdV biology. In the last decade, of 27 newly identified HAdVs, 19 were HAdV-Ds, suggesting the continuing evolution of new pathogens from species D. Analyses of 38 fully sequenced HAdV-D whole genomes identified homologous recombination of specific regions within the hexon, penton base, fiber, and E3 CR1 genes as the *major* mechanism behind HAdV-D evolution, a new finding (Robinson et al., 2013a; Singh et al., 2013). Stereotypical reductions in GC content at the junction of conserved and hypervariable regions, along with Chi-like sequence motifs (also a new finding), appear likely to augment the intrinsic tendency of HAdV to undergo homologous recombination *in vivo* (Lee et al., 2018).

Recently, the whole genome sequences of 85 HAdVs from archives and current collections were determined, including both historical and circulating strains, respectively (Ismail et al., 2018). Of these, 3 novel recombinants within HAdV-B and 15 within HAdV-D were identified. Only two of the 15 HAdV-Ds were found to contain novel genes (penton base and fiber); these were subsequently typed as HAdV-D71 and 72. Isolates of HAdV-D53 and HAdV-D58, two novel genotypes recently recognized, were also identified, adding confidence in their clinical importance. Fully genotyped HAdVs now number 90, with more awaiting type numbers, and the scientific community has a 10-fold larger database of unique HAdV genomes than available only 15 years ago. Published and validated ENCODE methodologies can now be applied, and comparisons made across disparate HAdV genomes. We suggest that the HAdV genome contains previously uncharacterized functional elements, and that every HAdV protein has pleiotropic interactions. Current technologies should afford a wave of new and important discoveries that may lead to needed therapies against adenoviral diseases.

## Author contributions

All authors listed have made a substantial, direct and intellectual contribution to the work, and approved it for publication.

### Conflict of interest statement

The authors declare that the research was conducted in the absence of any commercial or financial relationships that could be construed as a potential conflict of interest.
