# Corrigendum: Adenoviromics: Mining the Human Adenovirus Species D Genome

**DOI:** 10.3389/fmicb.2018.03005

**Published:** 2018-12-04

**Authors:** Ashrafali M. Ismail, Ji Sun Lee, Jeong Yoon Lee, Gurdeep Singh, David W. Dyer, Donald Seto, James Chodosh, Jaya Rajaiya

**Affiliations:** ^1^Howe Laboratory, Massachusetts Eye and Ear, Harvard Medical School, Boston, MA, United States; ^2^Molecular Virology Laboratory, Korea Zoonosis Research Institute Jeonbuk National University, Jeonju, South Korea; ^3^Department of Cell and Systems Biology, University of Toronto, Toronto, ON, Canada; ^4^Department of Microbiology and Immunology, University of Oklahoma Health Sciences Center, Oklahoma City, OK, United States; ^5^Bioinformatics and Computational Biology Program, School of Systems Biology, George Mason University, Manassas, VI, United States

**Keywords:** adenovirus, genome, evolution, transcription factor, interactome

In the original article, there were some minor errors in Tables 1, 2 as published. Some HAdV species names were wrongly spelled and some species names were left out. In addition, we have also updated the correct year of publication for these types. The corrected Tables 1, 2 appear below. The authors apologize for this error and state that this does not change the scientific conclusions of the article in any way. The original article has been updated.

**Table 1:** Species and type designations for the 51 human adenovirus (HAdV) serotypes.

**Type**	**GenBank accession no**.	**Genome length**	**Year published**
HAdV-C1	AC_000017	36001	2004
HAdV-C2	AC_000007	35937	2003
HAdV-B3	AY599834	35345	2006
HAdV-E4	AY599837	35964	2006
HAdV-C5	AY601635	35931	2006
HAdV-C6	FJ349096	35758	2011
HAdV-B7	KP670856.2	35239	2016
HAdV-D8	AB448767	34980	2009
HAdV-D9	AJ854486	35083	2008
HAdV-D10	JN226746	35105	2013
HAdV-B11	AF532578	34794	2003
HAdV-A12	X73487	34125	1979
HAdV-D13	JN226747	35209	2013
HAdV-B14	JQ824845	34767	2012
HAdV-D15	KF268204	35100	2013
HAdV-B16	JN860680	35384	2011
HAdV-D17	HQ910407	35139	2011
HAdV-A18	GU191019	34177	2010
HAdV-D19	JQ326209	35153	2011
HAdV-D20	JN226749	35181	2013
HAdV-B21	AY601633	35382	2006
HAdV-D22	FJ619037	35152	2009
HAdV-D23	JN226750	35050	2013
HAdV-D24	JN226751	35166	2013
HAdV-D25	JN226752	35248	2013
HAdV-D26	EF153474	35152	2007
HAdV-D27	JN226753	35154	2013
HAdV-D28	FJ824826	35130	2010
HAdV-D29	JN226754	35214	2013
HAdV-D30	JN226755	35178	2012
HAdV-A31	AM749299	33763	2005
HAdV-D32	JN226756	35248	2013
HAdV-D33	JN226758	35131	2013
HAdV-B34	AY737797	34775	2004
HAdV-B35	AC_000019	34794	2004
HAdV-D36	GQ384080	35152	2010
HAdV-D37	AB448775	35215	2009
HAdV-D38	JN226759	35221	2013
HAdV-D39	JN226760	35152	2013
HAdV-F40	NC_001454	34214	1993
HAdV-F41	DQ315364.2	34188	2007
HAdV-D42	JN226761	35231	2013
HAdV-D43	JN226762	35012	2013
HAdV-D44	JN226763	35214	2013
HAdV-D45	JN226764	35154	2013
HAdV-D46	AY875648	35178	2006
HAdV-D47	JN226757	35106	2013
HAdV-D48	EF153473	35206	2007
HAdV-D49	DQ393829	35215	2006
HAdV-B50	AY737798	35385	2007
HAdV-D51	JN226765	35114	2013

**Table 2:** Species and molecular types of human adenovirus (HAdV) genotypes 52–90.

**HAdV type**	**^#^Name**	**GenBank accession no**.	**Genome length**	**Year published**
HAdV-G52	P52H52F52/2003/USA	DQ923122.2	34250	2007
HAdV-D53	P37H22F8/2005/DEU	FJ169625	34909	2009
HAdV-D54	P54H54F8/2000/JPN	AB333801	34920	2008
HAdV-B55	P14H11F14/2006/CHN	FJ643676	34755	2010
HAdV-D56	P56H15F9/2008/FRA	HM770721	35066	2011
HAdV-C57	P1H57F6/2001/RUS	HQ003817	35818	2011
HAdV-D58	P58H58F29/1996/ARG	HQ883276	35217	2011
HAdV-D59	P64H25F56/2007/USA	JF799911	35072	2012
HAdV-D60	P60H20F60/2009/CAN	HQ007053	35050	2013
HAdV-A61	P31H31F31/2004/JPN	JF964962	33776	2011
HAdV-D62	P62H62F62/1993/GBR	JN162671	35127	2014
HAdV-D63	P30H30F29/1959/USA	JN935766	35168	2012
HAdV-D64	P22H19F37/1993/USA	EF121005	35231	2012
HAdV-D65	P58H10F9/2004/BGD	AP012285	35172	2012
HAdV-B66	P66H7F3/1987/ARG	JN860676	35080	2012
HAdV-D67	P67H9F67/2005/BGD	AP012302	35075	2013
HAdV-B68	P16H3F16/2004/ARG	JN860678	35538	Unpublished
HAdV-D69	P53H15F69/1955/SAU	JN226748	35124	2013
HAdV-D70	P70H70F29/2014/DEU	KP641339	35186	2015
HAdV-D71	P9H20F71/1987/DEU	KF268207	35192	2013
HAdV-D72	P72H30F72/1985/DEU	KF268335	34553	2013
HAdV-D73	P67H45F27/2015/DEU	KY618676	35190	2017
HAdV-D74	P70H74F51/2015/DEU	KY618677	35155	2017
HAdV-D75	P75H26F29/2015/DEU	KY618678	35104	2017
HAdV-B76	P21H21F16/DEU	KF633445	35586	2013
HAdV-B77	P35H34F7/1985/DEU	KF268328	34653	2013
HAdV-B78	P11H11F7/2000/ARG	KT970440	34881	Unpublished
HAdV-B79	P11H34F11/2015/JPN	LC177352	34779	2017
HAdV-D80	P19,23H28F22/2014/DEU	TBA	34909	Unpublished
HAdV-D81	P65H48F60/2012/JPN	AB765926.1	35198	2014
HAdV-D82	P56H15F37/2011/JPN	LC066535.1	35122	Unpublished
HAdV-D83	P83H9F15/2010/PAR	KX827426.1	35207	2017
HAdV-D84	P43H17F84/2011/PAN	MF416150	35257	2017
HAdV-D85	P37H19F8/2015/JPN	LC314153	35203	2018
HAdV-D86	P9H25F25/1978/SWE	TBA	35147	Unpublished
HAdV-D87	P9H15F25/1967/USA	MF476841	35159	Unpublished
HAdV-D88	P88H15F9/1963/USA	MF476842	35115	Unpublished
HAdV-C89	P89H2F2/2015/DEU	TBA	35998	Unpublished
HAdV-D90	P33H27F67/2017/BGD	TBA	34207^*^	Unpublished

# Name indicates molecular type (P, penton base; H, hexon; F, fiber)/year of isolation/country of isolation.

TBA: GenBank accession number, to be assigned.

* *Metagenomics project, missing the inverted terminal repeat sequences*.

## Conflict of Interest Statement

The authors declare that the research was conducted in the absence of any commercial or financial relationships that could be construed as a potential conflict of interest.

